# Variation in practice and outcomes after inguinal hernia repair: a nationwide observational study

**DOI:** 10.1186/s12893-020-01030-0

**Published:** 2021-01-20

**Authors:** Carmen S. S. Latenstein, Floris M. Thunnissen, Mitchell Harker, Stef Groenewoud, Mark W. Noordenbos, Femke Atsma, Philip R. de Reuver

**Affiliations:** 1 Department of Surgery, Radboud University Medical Centre Nijmegen, Geert Grooteplein 10, P.O. Box 9101, 6500 HB Nijmegen, The Netherlands; 2 Scientific Institute for Quality of Healthcare (IQ Healthcare), Radboud University Medical Centre, Nijmegen, The Netherlands

**Keywords:** Hospital variation, Inguinal hernia, Contributing factors, Clinical outcome

## Abstract

**Background:**

Inguinal hernia repair has often been used as a showcase to illustrate practice variation in surgery. This study determined the degree of hospital variation in proportion of patients with an inguinal hernia undergoing operative repair and the effect of this variation on clinical outcomes.

**Methods:**

A nationwide, longitudinal, database study was performed in all hospitals in the Netherlands between 2013 and 2015. Patients with inguinal hernias were collected from the Diagnosis-Related-Group (DRG) database. The case-mix adjusted operation rate in patients with a new DRG determines the observed variation. Hospital variation in case-mix adjusted inguinal hernia repair-rates was calculated per year. Clinical outcomes after surgery were compared between hospitals with high and low adjusted operation-rates.

**Results:**

In total, 95,637 patients were included. The overall operation rate was 71.6%. In 2013–2015, the case-mix adjusted performance of inguinal hernia repairs in hospitals with high rates was 1.6–1.9 times higher than in hospitals with low rates. Moreover, in hospitals with high adjusted rates of inguinal hernia repair the time to surgery was shorter, more laparoscopic procedures were performed, less emergency department visits were recorded post-operatively, while more emergency department visits were recorded when patients were treated conservatively compared to hospitals with low adjusted operation rates.

**Conclusion:**

Hospital variation in inguinal hernia repair in the Netherlands is modest, operation-rates vary by less than two-fold, and variation is stable over time. Hernia repair in hospitals with high adjusted rates of inguinal hernia repair are associated with improved outcomes.

## Background

Inguinal hernia repair is one of the most performed surgical procedures worldwide and is previously used to illustrate surgical practice variation [1–3]. In general, practice variation relates to differences in quality and inequity in the delivery of health care [4–6]. In hernia surgery, a conservative approach is a reasonable option for patients with a minimally symptomatic inguinal hernia, but this watchful-waiting policy is not applied by all hernia specialists [7].

In the United States, surgeons perform 770,000 hernia repairs annually, 28,000 procedures are performed in the Netherlands each year [2, 3, 8]. Practice variation in terms of procedures per 100,000 inhabitants show differences between countries: from 69/100,000 in Korea, to 177/100,000 in the Netherlands, 197/100,000 in the United States, and 269/100,000 in Austria [9]. Several European reports show that whether a patient undergoes a general surgical procedure or not, depends heavily on the region or hospital [1, 10, 11]. However, longitudinal, nationwide studies in patients with inguinal hernia are scarce. Quantifying practice variation in hernia surgery is important to identify the cause of variation, to find leads for improvement of care (e.g. less surgeries in asymptomatic patients), and to find leads reduce potential unwarranted variation [12].

This study aimed to determine longitudinal hospital variation in inguinal hernia repair in a nationwide cohort in the Netherlands, while adjusting for case-mix. Furthermore, the clinical outcomes and hospital characteristics were compared between hospitals with low or high rates of inguinal hernia repair.

## Methods

### Study population and data

For the present study, data of patients with an inguinal hernia from 2013, 2014 and 2015 were extracted from a routinely collected nationwide database with hospital data. In the Netherlands, hospital care reimbursements are based on Diagnosis Related Groups (DRGs). These DRGs consist of an average of healthcare costs for a combination of various treatments. These do not give information about the actual care provided. Our database included both DRGs and healthcare activities, so we could use the actual provided care in our analyses [8]. These healthcare activities (such as surgery or post-operative emergency department visits) are registered by all Dutch hospitals. At time of the study, the coverage was 90% for 2013, 90% for 2014, and 80% for 2015 (due to administrative delays in registries). This research was performed in accordance with the ethical standards of the Helsinki Declaration of 1975. Also, we followed the Strengthening the Reporting of Observational studies in Epidemiology (STROBE) guideline and the Reporting of studies conducted using observational routinely-collected health data (RECORD) statement [13, 14]. The present study did not require approval from an ethics committee in the Netherlands; moreover, registration in the DRG-database (with both DRGs as well as the healthcare activities) did not require written informed consent.

### Data collection

All newly diagnosed patients were selected based on diagnosis codes (hernia femoralis/inguinalis, Dutch code: 303–121 and hydrocele communicans, Dutch code: 306-67). In the database, detailed information was available about actual performed health care procedures. Two groups were formed, a group of patients who received surgical treatment and a group who received conservative treatment. The following healthcare procedures were identified as surgical treatment: laparoscopic inguinal hernia repair, laparoscopic inguinal hernia recurrence repair, open inguinal hernia repair, open femoral hernia repair, open inguinal hernia recurrence repair, and laparoscopic femoral hernia repair. Patients who received surgery were assigned to the hospital where the surgery took place. Conservatively treated patients were assigned to the last visiting hospital.

Patient characteristics and type of treatment included sex, age, socio economic status (SES)-score, type of surgery (open or laparoscopically), and time interval between diagnosis and surgery [15]. In the surgical treatment group, 30-day follow-up included emergency department visits, readmissions, and reoperations. Only emergency department visits within 30 days after diagnosis were assessed for the conservative treatment group.

Type of hospital (general, academic, private) was also available in the database. Hospital characteristics, for the entire hospital and more specific for the department of surgery, were collected from the register of the Dutch Ministry of Healthcare, Welfare and Sports; summarizing hospital characteristics from 2014 [16, 17].

### Study outcomes

The operation-rate was defined as the proportion of patients with a hernia undergoing surgical repair. We performed case-mix adjustments (sex, age, SES-score) for the operation-rate. The primary outcome of the study was the variation in case-mix adjusted operation-rates between hospitals. First, the variation in case-mix adjusted operation-rate per 1,000 patients was calculated between all Dutch hospitals. In an additional analysis, academic hospitals and private clinics were excluded to better asses the influence of hospitals on variation and for the selection of a more homogenous patient population, because academic hospitals do not perform elective hernia repairs in the Netherlands. Subsequently, we compared clinical outcomes of hospitals with a low and high case-mix adjusted operation-rate. Finally, hospital characteristics were compared of hospitals with low and high case-mix adjusted operation-rates.

### Statistical analysis

#### Descriptives

Patient characteristics were calculated for all individual patients diagnosed with an inguinal hernia. Age was presented as mean with standard deviation; sex and surgical treatment were presented as percentages.

#### Hospital variation

We analyzed hospital variation between all types of hospitals and only between general hospitals. All analyses were based on individual patient data and not on population-based data. Hospitals were excluded if they had less than 30 newly diagnosed patients with inguinal hernia, or if they performed less than five surgical operations of interest per year.

First, the case-mix adjusted operation-rate per hospital was calculated. The calculation of the case-mix adjusted operations per 1,000 hernia patients consisted of two steps: a logistic regression with surgery/conservative treatment as outcome and age, sex and SES as covariates was performed to assess the expected operations per hospital. Subsequently, we assessed the ratio of performed to expected number of operations per 1,000 hernia patients and multiplied by the national average of inguinal hernia repairs.

Second, to express the degree of hospital variation, two types of factor scores were calculated [18, 19]. Factor scores show by which factor the highest scores differ from the lowest scores and were calculated by dividing the mean of the three hospitals with the highest adjusted operation rates by the mean of the three hospitals with the lowest adjusted operation rates. Additionally, to exclude the influence of outliers, factor scores were based on the 95th percentile and 5th percentile of the distribution of case-mix adjusted operation rates by dividing the 95th percentile by the adjusted rate in the 5th percentile. International literature showed that a factor score below 2.0 is modest [20]. This means that a patient visiting the hospital in the 95th percentile has a 2 times higher chance of undergoing treatment compared to a hospital in the 5th percentile. Factor scores were calculated between all hospitals and between general hospitals only. Hospital variation was presented in bar charts.

Comparing general hospitals with a low or high adjusted rate of inguinal hernia repair.

Academic hospitals and private clinics were excluded to compare clinical outcomes and hospital characteristics between general hospitals with a low or high case-mix-adjusted operation-rate. A hospital was defined as “hospital with a low adjusted rate of inguinal hernia repair” when a hospital appeared in the lowest 20th percentile of the case-mix adjusted operation-rates in all three subsequent years (2013, 2014 and 2015). A hospital was defined as “hospital with a high adjusted rate of inguinal hernia repair” when a hospital appeared in the highest 20th percentile of the case-mix adjusted operation-rate in all three subsequent years.

Days to operation were presented in means with standard deviation. Other clinical outcomes, and patient- and treatment characteristics were presented as percentages. Hospital characteristics were presented as means per hospital and percentages for hospitals with a low adjusted rate of inguinal hernia repair and hospitals with a high adjusted rate of inguinal hernia repair. Analyses of continuous data were done using Student T-test for normally distributed data and Mann–Whitney U-test for skewed data. Dichotomous data were analyzed using chi-square test. Associations with a p-value less than 0.05 were considered statistically significant. Analyses were performed with R version 3.5.1 and SPSS version 22.

## Results

### Patient population

The number of inguinal hernia diagnoses in 2013, 2014, and 2015 were 33,181, 33,146, and 29,310, respectively. Six hospitals were excluded due to low patient or operation numbers in 2013, 2014, and 2015. The patient characteristics are summarized in Table 1.

**Table 1:** Patient characteristics and hospital variation factor scores

	All hospitals	General hospitals
2013		
Hospitals, n	85	74
Patients, n	33,181	31,419
Sex male, n (%)	29,915 (90.2)	28,370 (90.3)
Age, mean (SD)	59.6 (15.9)	59.6 (15.8)
Surgical treatment, n (%)	24,010 (72.4)	22,811 (72.6)
Factor score^a^	1.88 (2.47)^b^	1.59 (2.01)^b^
2014		
Hospitals, n^c^	79	69
Patients, n	33,146	31,523
Sex male, n (%)	29,779 (89.8)	28,361 (90.0)
Age, mean (SD)	59.9 (15.9)	60.0 (15.8)
Surgical treatment, n (%)	23,724 (71.6)	22,634 (71.8)
Factor score^a^	1.66 (2.72)^b^	1.39 (1.58)^b^
2015		
Hospitals, n	68	59
Patients, n	29,310	27,740
Sex male, n (%)	26,545 (90.6)	25,130 (90.6)
Age, mean (SD)	60.4 (15.9)	60.5 (15.8)
Surgical treatment, n (%)	20,801 (71.0)	19,814 (71.4)
Factor score^a^	1.55 (3.08)^b^	1.38 (1.45)^b^

^a^The factor score is a measure to describe the degree of hospital variation. It shows by which factor the highest scores differ from the lowest scores. Factor scores were based on the 95th percentile and 5th percentile of the distribution of case-mix adjusted inguinal hernia repair rates. The case-mix adjusted inguinal hernia repair-rate of the 95th percentile was divided by the case-mix adjusted inguinal hernia repair-rate of the 5th percentile. Additionally, the factor score was calculated by dividing the mean of the case-mix adjusted inguinal hernia repair rates of the three hospitals with the highest adjusted rates by the mean of the three hospitals with the lowest adjusted rates

^b^95th percentile/5th (top 3/bottom 3)

^c^The number of hospitals went down each year due to fusion of hospitals. At time of the study, the coverage was 90% for 2013, 90% for 2014, and 80% for 2015 (due to administrative delays in registries). Therefore, the number of patients is lower in 2015

### Variation between all hospitals

**Fig. 1:**
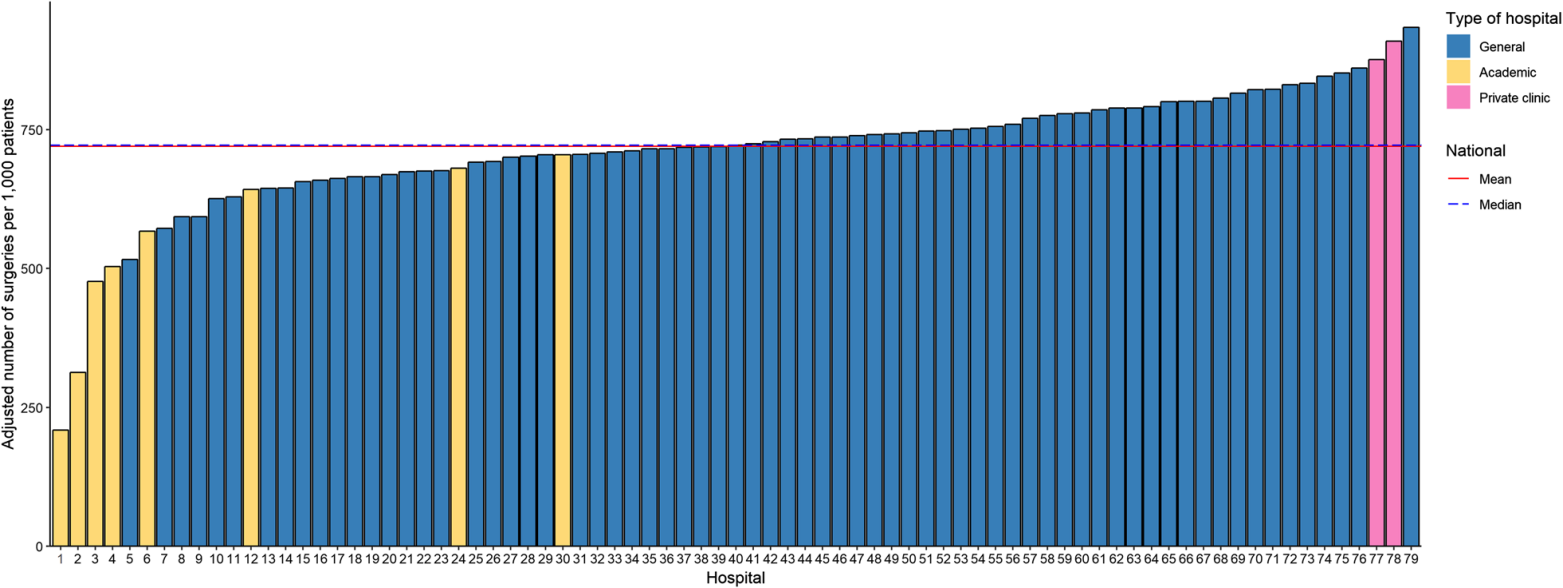
Hospital variation in inguinal hernia repairs in all Dutch hospitals. Case-mix adjusted operations per 1,000 patients per hospital. Adjustments were made for sex, age, and social-economic status. Horizontal lines illustrate the mean/median case-mix adjusted operations per 1000 patients. Each bar represents one hospital. Yellow bars implicate academic hospitals, pink bars implicate private clinics and blue bars implicate general hospital.

The case-mix adjusted repairs per 1,000 hernia patients per hospital in 2014 are illustrated in Fig. 1. In 2014, the 95th percentile/5th percentile factor score was 1.66 (top 3/bottom 3; 2.72). In other words, the number of case-mix adjusted operations per 1,000 patients was 850 in the 95th percentile and 511 in the 5th percentile (850/511 = 1.66). In 2013 and 2015 factor scores were 1.88 (top 3/bottom 3; 2.47) and 1.55 (top 3/bottom 3; 3.08), respectively (Table 1).

### Variation between general hospitals

Eight academic hospitals and two private clinics were excluded. In 2014, the 95th percentile/5th percentile factor scores between general hospitals was 1.39 (top 3/bottom 3; 1.58). The factor scores for 2013 and 2015 were 1.59 (top 3/bottom 3; 2.01) and 1.38 (top 3/bottom 3; 1.45), respectively (Table 1).

### Differences between hospitals with low and high adjusted inguinal hernia repair rates

Four general hospitals met the definition for a hospital with a low adjusted rate of inguinal hernia repair (case-mix adjusted operation-rate in the lowest 20th percentile in three consecutive years) and three general hospitals met the definition for a hospital with a high adjusted rate of inguinal hernia repair (highest 20th percentile in three consecutive years).

In hospitals with high adjusted rates of inguinal hernia repair, an inguinal hernia was performed earlier after diagnosis (26 days vs. 45 days, p = 0.016), more often by laparoscopy (66% vs. 34%, p < 0.001), postoperative outcomes were associated with less emergency department visits within 30 days after operation (2.4% vs. 4.4%, p = 0.008), and outcomes of conservatively treated patients were associated with more emergency department visits within 30 days (4.9% vs. 1.5%, p = 0.004), as compared to hospitals with low adjusted rates of inguinal hernia repair (Table 2).

**Table 2:** Patient characteristics, treatment characteristics and clinical outcome of hospitals with low and high adjusted rates of inguinal hernia repair

Clinical outcome	Hospital		p-value
	Low^a^	High^b^	
Patients, n	1811	1367	
Sex male, n (%)	1622 (89.5)	1244 (91.0)	
Age, mean (SD)	61.14 (3.2)	60.26 (1.0)	
Conservative treatment	*N* = 684	*N* = 206	
Emergency department visit < 30 days after diagnosis—n (%)	10 (1.5)	10 (4.9)	*0.004*
Operative treatment	*N* = 1127	*N* = 1161	
Laparoscopic operation—n (%)	384 (34.1)	766 (66.0)	*< 0.001*
Days to operation—weighted mean (SD)	45.37 (6.1)	26.33 (8.7)	*0.016*
Emergency department visit < 30 days after diagnosis—n (%)	14 (1.2)	13 (1.1)	0.786
Emergency department visits < 30 days after operation—n (%)	50 (4.4)	28 (2.4)	*0.008*
Readmission < 30 days after operation—n (%)	14 (1.2)	23 (2.0)	0.161
Reoperation < 30 days after operation—n (%)	0	0	NA

^a^A general hospital was defined as “hospital with a low adjusted inguinal hernia repair rate” when a hospital appeared in the lowest 20th percentile of the distribution of the adjusted number of surgeries per 1000 patients in all three subsequent years (2013, 2014 and 2015)

^b^A general hospital was defined as “hospital with a high adjusted inguinal hernia repair rate” when a hospital appeared in the highest 20th percentile of the distribution of the adjusted number of surgeries per 1000 patients in all three subsequent years (2013, 2014 and 2015)

In hospitals with low adjusted rates of inguinal hernia repair, most treatment of the medical conditions were reimbursed by a fixed price (Table 3). Hospitals with low adjusted rates of inguinal hernia repair were more often teaching hospitals, while hospitals with high adjusted rates of inguinal hernia repair were non-teaching hospitals. Other hospital characteristics, such as annual revenue, bed capacity, operation rooms, number of surgeons, and number of medical specialists and in what way they receive money were comparable between hospitals with high and low adjusted rates of inguinal hernia repair.

**Table 3:** Hospital characteristics of hospitals with low and high adjusted rates of inguinal hernia repair

Hospital characteristics	Hospital		p-value
	Low^a^	High^b^	
Surgical teaching hospital^c^—yes/no	3/1	1/2	
Another hospital, same city—yes/no	0/4	0/3	
Characteristics per hospital			
Annual revenue—mean (millions, €)	230	167	0.301
Newly registered medical conditions, mean	266,193	178,311	0.302
Fixed price, %	22.9	6.25	*< 0.001*
Not fixed/free price, %	77.1	93.7	
Operation rooms, mean	14	9	0.172
Bed capacity, mean	514	412	0.421
Staff, mean	2694	2326	0.582
Medical specialist, mean	196	151	0.258
Salaried, %	33.1	24.5	0.079
Self-employed, %	66.9	75.4	
Surgeons, mean	12	10	0.404

^a^A general hospital was defined as “hospital with a low adjusted inguinal hernia repair rate” when a hospital appeared in the lowest 20th percentile of the distribution of the adjusted number of surgeries per 1000 patients in all three subsequent years (2013, 2014 and 2015)

^b^A general hospital was defined as “hospital with a high adjusted inguinal hernia repair rate” when a hospital appeared in the highest 20th percentile of the distribution of the adjusted number of surgeries per 1000 patients in all three subsequent years (2013, 2014 and 2015)

^c^Involvement of surgical trainees in surgery

## Discussion

This nationwide database study shows that practice variation in inguinal hernia repair is modest in the Netherlands. Operation-rates vary by less than two-fold, and variation is stable over the years 2013 to 2015. A more thorough analysis illustrates that the type of hospital (academic, teaching, or private) is the most relevant factor contributing to the observed variation.

An addition to previous reports on practice variation in hernia surgery is the present finding that adjusted rates in surgery in general hospitals are associated with the type of financial reimbursement for diagnosis and the percentage of self-employed staff. These non-clinical factors related to variation may not only contribute to practice variation in hernia surgery, but also in carotid endarterectomies, lumbar hernia repair, or in hip or knee replacements for osteoarthritis. The reported practice variation in surgery is significantly higher in these conditions with operation-rates varying over a ten-fold [10, 11, 19, 21]. The present study also shows that surgical outcomes in hospitals with high adjusted rates in surgery seem better compared to hospitals with low adjusted rates. In the hospitals with high adjusted rates, time to surgery is shorter, repairs are more often performed laparoscopically, and we observed less emergency department visits within 30 days after the operation.

Since 2003, the Dutch Society of Surgery has implemented evidence-based guidelines for an inguinal hernia repair [22]. Several Dutch RCTs and observational studies serve as clinical evidence in this guideline. Multicenter research contributed to the nationwide implementation and guideline adherence [23–25]. Comprehensive communication about guideline content to surgeons in training and the practicing community made the Dutch hernia service more uniform [26]. These guidelines contributed to improved decision-making for surgery from the surgeon’s perspective. But additionally, the introduction of a shared decision-making strategy and online decision aids may have helped to increase a watchful-waiting policy and reduce hospital variation [27]. The E-valuAID trial currently investigates if a shared decision-making strategy and online decision aids in patients with an inguinal hernia are cost-effective (The Netherlands Trial Register NL8318). The outcome of this trial will inform clinicians and policymakers whether decision aids indeed improve patient-reported outcomes and do contribute to reduced operation rates.

The Dutch guidelines advocate laparoscopic hernia repair if sufficient experience is present as this procedure has shown to be safe and cost-effective [28]. This policy is reflected in the higher laparoscopy rate in hospitals with high adjusted operation rates [22]. This policy is reflected in the higher laparoscopy rate in hospitals with high adjusted operation rates. Birkmeyer et al. summarized several methods to reduce variation in the use of surgery [4]. After a thorough review of the literature, the authors concluded that operation rates are affected by both system-level changes, and interventions directed at the doctor-patient relationship. They also found that appropriate rates of surgical treatment are achieved by clear, valid evidence about indications. Finally, their paper addresses that formal aids for decision making affect rates of surgery substantially, usually (but not always) reducing the rate of surgery. It is of interest that these three aspects all seem to apply for inguinal hernia repair in the Netherlands.

A strength of the present study is that we were able to include nationwide, longitudinal data. Additionally, practice variation data was adjusted for sex, age and social-economic status. Moreover, we performed secondary analyses only on general hospitals, as academic hospitals and private clinics both treat a selected group of patients. Inherent to the data source, the database lacked specified clinical data on patient characteristics, severity of symptoms, comorbidity, disease prevalence, percentage of femoral hernias, incarcerated hernias, double-sided hernias, and patient-reported outcomes, which are limitations of this study. Moreover, we only measured hospital variation in three consecutive years and were not able to assess practice variation among individual surgeons.

In summary, hospital variation in inguinal hernia repair in the Netherlands is modest. Nationwide research and comprehensive communication may have contributed to an uniform Dutch hernia service.

## Conclusions

Hospital variation in inguinal hernia repair in the Netherlands is modest, operation-rates vary by less than two-fold, and variation is stable over time. Hernia repair in hospitals with high adjusted rates of inguinal hernia repair are associated with improved outcomes. This research is relevant because high practice variation rates relates to differences in quality and inequity in health care.

## Data Availability

Data, methods and study material available: Yes. Data types: De-identified hospital data. How to access data: Philip de Reuver (Philip.dereuver@radboudumc.nl).

## Abbreviations

DRG: Diagnosis-Related-Group database
STROBE: Strengthening the reporting of observational studies in epidemiology guideline
RECORD: Reporting of studies conducted using observational routinely-collected health data
SES: Socio economic status
RCTs: Randomized controlled trials
